# Exosomal miR-9-5p derived from iPSC-MSCs ameliorates doxorubicin-induced cardiomyopathy by inhibiting cardiomyocyte senescence

**DOI:** 10.1186/s12951-024-02421-8

**Published:** 2024-04-20

**Authors:** Huifeng Zheng, Xiaoting Liang, Baojuan Liu, Xinran Huang, Ying Shen, Fang Lin, Jiaqi Chen, Xiaoyan Gao, Haiwei He, Weifeng Li, Bei Hu, Xin Li, Yuelin Zhang

**Affiliations:** 1 Department of Emergency Medicine, Guangdong Provincial People’s Hospital (Guangdong Academy of Medical Sciences), Southern Medical University, Guangzhou, Guangdong China; 2 Department of Intensive Care Unit, Chongqing General Hospital, Chongqing, China; 3 Translational Medical Center for Stem Cell Therapy and Institute for Regenerative Medicine, Shanghai East Hospital, Tongji University School of Medicine, Shanghai, China; 4 Shanghai Heart Failure Research Center, Shanghai East Hospital, Tongji University School of Medicine, Shanghai, China

**Keywords:** Exosomes, Mesenchymal stem cells, Doxorubicin, Cardiomyopathy, Senescence

## Abstract

Doxorubicin (DOX) is a chemotherapeutic agent widely used for tumor treatment. Nonetheless its clinical application is heavily limited by its cardiotoxicity. There is accumulated evidence that transplantation of mesenchymal stem cell-derived exosomes (MSC-EXOs) can protect against Dox-induced cardiomyopathy (DIC). This study aimed to examine the cardioprotective effects of EXOs isolated from human induced pluripotent stem cell-derived MSCs (iPSC-MSCs) against DIC and explore the potential mechanisms. EXOs were isolated from the cultural supernatant of human BM-MSCs (BM-MSC-EXOs) and iPSC-MSCs (iPSC-MSC-EXOs) by ultracentrifugation. A mouse model of DIC was induced by intraperitoneal injection of Dox followed by tail vein injection of PBS, BM-MSC-EXOs, or iPSC-MSC-EXOs. Cardiac function, cardiomyocyte senescence and mitochondrial dynamics in each group were assessed. In vitro, neonatal mouse cardiomyocytes (NMCMs) were subjected to Dox and treated with BM-MSC-EXOs or iPSC-MSC-EXOs. The mitochondrial morphology and cellular senescence of NMCMs were examined by Mitotracker staining and senescence-associated-β-galactosidase assay, respectively. Compared with BM-MSC-EXOs, mice treated with iPSC-MSC-EXOs displayed improved cardiac function and decreased cardiomyocyte mitochondrial fragmentation and senescence. In vitro, iPSC-MSC-EXOs were superior to BM-MSC-EXOs in attenuation of cardiomyocyte mitochondrial fragmentation and senescence caused by DOX. MicroRNA sequencing revealed a higher level of miR-9-5p in iPSC-MSC-EXOs than BM-MSC-EXOs. Mechanistically, iPSC-MSC-EXOs transported miR-9-5p into DOX-treated cardiomyocytes, thereby suppressing cardiomyocyte mitochondrial fragmentation and senescence via regulation of the VPO1/ERK signal pathway. These protective effects and cardioprotection against DIC were largely reversed by knockdown of miR-9-5p in iPSC-MSC-EXOs. Our results showed that miR-9-5p transferred by iPSC-MSC-EXOs protected against DIC by alleviating cardiomyocyte senescence via inhibition of the VPO1/ERK pathway. This study offers new insight into the application of iPSC-MSC-EXOs as a novel therapeutic strategy for DIC treatment.

**Graphical Abstract:**

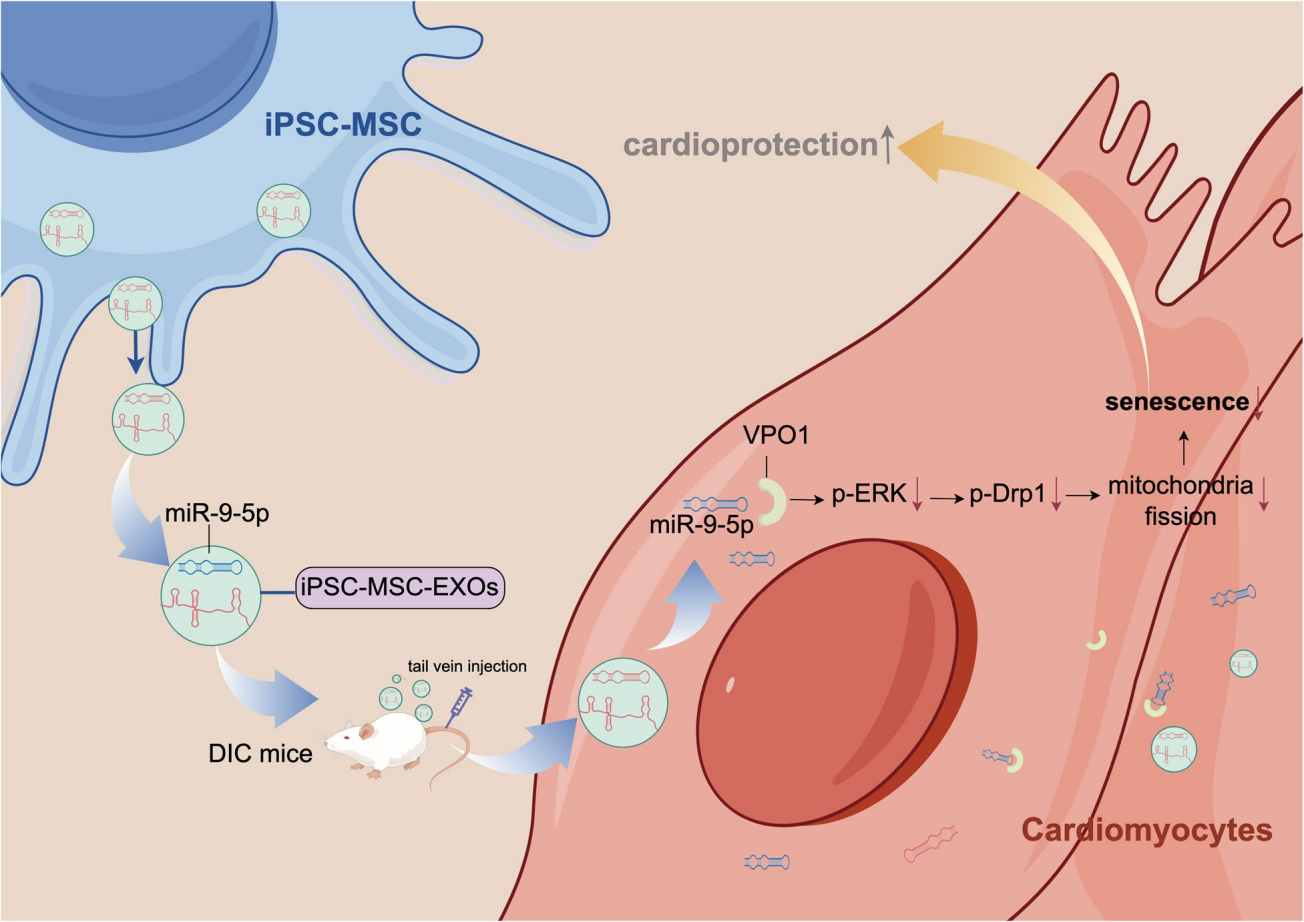

**Supplementary Information:**

The online version contains supplementary material available at 10.1186/s12951-024-02421-8.

## Introduction

Doxorubicin (DOX) is a broad-spectrum chemotherapeutic drug widely used in various types of human malignant tumors. Nonetheless its clinical application is severely hampered by its cumulative and dose-dependent cardiotoxicity including arrhythmia, cardiomegaly and cardiomyopathy [1, 2]. Apart from dexrazoxane, the only protective agent approved by the FDA, there is no cure for Dox-induced cardiomyopathy (DIC) due to the complicated molecular mechanisms involved [3]. Although the potential mechanisms underlying DIC are not fully understood, excessive production of reactive oxygen species (ROS), apoptosis, ferroptosis and autophagy have been reported to be closely linked to the disease [4–6]. There is also recent evidence that cardiomyocyte senescence plays a critical role in DIC development [7–9]. It has been well documented that DOX treatment impaired cardiac function in mice via stimulation of cardiomyocyte senescence, and elimination of senescent cells with the senolytic Navitoclax significantly improved heart function [10].

Although the precise mechanisms remain to be illustrated, multiple studies have established that an imbalance in mitochondrial dynamics that are regulated by mitochondrial fission and fusion contributes to several types of cellular senescence including that of cardiomyocytes [11, 12]. Mitochondria undergo mitochondrial fission, regulated by mitochondrial fission 1 protein (Fis1) and dynamin-related protein 1 (Drp1), as well as mitochondrial fusion, controlled by optic atrophy protein 1 (OPA1) and Mitofusin1/2 (Mfn1/2), to maintain mitochondrial homeostasis [13]. Disruption of this balance of mitochondrial dynamics directly affects mitochondrial dysfunction, leading to cellular senescence [14]. Our previous study also showed that ischemia induced cardiomyocyte senescence by activating mitochondrial fission, accelerating heart dysfunction in a mouse model of myocardial infarction [15]. Interestingly, DOX treatment resulted in an increased tendency of mitochondrial fission in cardiomyocytes, manifested by a reduced size of mitochondria, leading to heart dysfunction [16]. Nonetheless it is unclear whether DOX induces cardiomyocyte senescence via regulation of mitochondrial dynamics.

Over the past decades, there has been increasing evidence that adult mesenchymal stem cell-derived exosomes (MSC-EXOs) exert beneficial effects on DIC by transferring a vast array of useful biological components including microRNAs (miRNAs), long noncoding RNAs (lncRNA) and proteins [17, 18]. In a mouse model of DIC, transplantation of MSC-EXOs resulted in recovery of cardiac function via delivery of LncRNA-NEAT1. These effects were further augmented by macrophage migration inhibitory factor pretreated-MSC-EXOs [19]. Currently, adult bone marrow derived MSCs (BM-MSCs) have been the most investigated cell source in experimental studies and clinical trials. Nevertheless their function declines with aging or long-term culture in vitro, thus directly impairing the function of MSC-EXO [20]. Our group has recently derived functional MSCs from induced pluripotent stem cells (iPSC-MSCs). Compared with BM-MSCs, iPSC-MSCs exhibited similar characteristics while demonstrating increased proliferation, enhanced immune privilege, and reduced batch-to-batch variation [21, 22]. More importantly, we revealed that iPSC-MSCs are superior to BM-MSCs in their attenuation of DIC due to their stronger paracrine action and higher mitochondrial transfer capacity [23, 24]. These findings prompted us to investigate the cardioprotective effects of iPSC-MSC-EXOs against DIC and explore the potential molecular mechanisms. In the current study, we investigated the cardioprotective effects and mechanisms of iPSC-MSC-EXO-derived exosomal miR-9-5p on DIC.

## Methods and materials

### Cell culture

BM-MSCs and iPSC-MSCs were routinely cultured as described previously [23]. Briefly, MSCs were cultured in DMEM containing 10% fetal calf serum (FBS, GIBCO, 10270-106), 10 ng/mL epidermal growth factor (PeProTech, AF-100-15) and 5 ng/mL basic fibroblast growth factor (PeProTech, 100-18B). BM-MSCs at passage 3 ~ 4 and iPSC-MSCs at passage 6 ~ 8 were used in this study. Neonatal mouse cardiomyocytes (NMCMs) were isolated from heart tissue of neonatal mice (0- to 1-day-old) as described previously [24], then cultured at 37 °C in Claycomb Medium (Sigma, 51800) supplemented with 10% FBS.

### Isolation and identification of MSC-EXOs

BM-MSC-EXOs and iPSC-MSC-EXOs were isolated from BM-MSCs and iPSC-MSCs and characterized as reported previously [15]. Briefly, 1 × 10^6^ BM-MSCs or iPSC-MSCs were cultured in a 10-cm culture dish for 24 h and the culture medium then replaced with DMEM containing 10% exosome-depleted FBS (Systems Biosciences, EXO-FBS-250A-1). After a further 48 h culture, the supernatant was harvested and EXOs isolated using anion exchange chromatography. Next, MSC-EXOs were suspended in PBS and their concentration measured with a BCA assay kit (Thermo, 231227). To knockdown the level miR-9-5p in iPSC-MSC-EXOs, iPSC-MSCs were transfected with 50 nM miR-9-5p inhibitor and miR-9-5p^KD^-iPSC-MSC-EXOs isolated. The size and distribution of MSC-EXOs was assessed by Nanoparticle tracking analysis (NTA). The morphology of MSC-EXOs was determined by transmission electron microscopy (TEM) and exosomal surface markers determined by Western blotting.

### Internalization of MSC-EXOs

To examine the uptake of MSC-EXOs by cardiomyocytes, MSC-EXOs were labeled with Dil (Beyotime, C1036) and then co-cultured with NMCMs for 24 h. After washing twice with PBS, NMCMs were fixed in 4% paraformaldehyde for 15 min and then stained with DAPI (Beyotime, C1005) for 15 min. Finally, NMCMs with Dil-labeled-MSC-EXOs were photographed under a confocal microscope.

### SA-β-gal (senescence-associated β-galactosidase) assay

NMCM senescence was assessed by SA-β-gal staining according to the manufacturer's instructions (Beyotime, #C0602). Briefly, NMCMs were cultured in a 6-well culture plate and treated with PBS, 10 μg/mL BM-MSC-EXOs or 10 μg/mL iPSC-MSC-EXOs under 1 μM DOX (MCE, HY-15142) challenge for 72 h. Next, cells were stained overnight with SA-β-gal solution at 37 °C without CO_2_. Subsequently, SA-β-gal positive NMCMs, evidenced by blue color were photographed under a microscope from five different fields of view. The percentage of senescent NMCMs was determined as the ratio of SA-β-gal positive NMCMs to total number of NMCMs.

### MitoTracker staining

To detect the morphology of mitochondria in NMCMs, MitoTracker staining was performed. Briefly, NMCMs were seeded on 24-well plates with cover slides and the different treatments described above administered. Next, NMCMs were washed with PBS and incubated for 20 min at room temperature with DMEM containing 20 nM MitoTracker Green FM (Invitrogen, M7514). Subsequently, after washing with PBS, the stained NMCMs were randomly imaged from six fields and at least 300 NMCMs were counted in each group. Finally, the ratio of NMCMs with fragmented mitochondria to total number of NMCMs was calculated.

### Transfection of miR-9-5p mimic or inhibitor

miR-9-5p mimic, miR-9-5p inhibitor and miR-Control were purchased from GenePharma Co., Ltd (Shanghai, China). Briefly, 1 × 10^6^ iPSC-MSCs were seeded on a 10-cm culture dish and cultured for 24 h. Next, MSCs were transfected with 50 nM miR-9-5p mimic, inhibitor or miR-Control using Lipofectamine 2000 transfection reagent (Invitrogen, 11668027) and cultured for 48 h at 37 °C in a 5% CO_2_ incubator. Finally, the transfection efficiency was evaluated by qRT-PCR. The supernatant was harvested to collect EXOs according to protocols described above.

### Luciferase assay

The 3′-UTR of human VPO1 (Vascular peroxidase 1) containing the miR-9-5p target site or the mutation in the seed region of the miR-9-5p binding site was inserted into the pGL3 luciferase reporter vector (Promega, Madison, WI, USA). 293 T cells were co-transfected with the reporter plasmid (pGL3-VPO1-3′-UTR or mutant VPO1-3′-UTR vector) and miR-9-5p mimics or inhibitors or miR-Control by Lipofectamine 2000 (Invitrogen, 11668027). Finally, luciferase activity was examined at 48 h after transfection using a Dual-Luciferase Reporter Assay System Kit (E1910, Promega).

### Quantitative real-time PCR

Total RNA from NMCMs with or without different treatments, BM-MSC-EXOs and iPSC-MSC-EXOs was extracted with TRIzol reagent (Takara, 2270A). Reverse transcription was carried out using a PrimeScript RT Reagent Kit (Takara, RR037A). RT-PCR for miRNAs or VPO1 was determined using a One-Step TB Green® PrimeScript™ RT-PCR Kit according to the protocol (Takara, RR820A). GAPDH and U6 served as the internal reference. Relative expression of miRNAs and VPO1 mRNA was normalized and calculated by the 2 − ΔΔCt method.

### Exosomal miRNA sequencing

Total RNA from BM-MSC-EXOs and iPSC-MSC-EXOs was extracted using a miRNeasy® Mini kit (Qiagen, 217004). The miRNA was sequenced using Illumina HiSeqTM 2500 (Genedenovo Co. Ltd, Guangzhou, China) as reported previously [15]. Raw reads were normalized and the expression of miRNAs analyzed to detect significant differences between BM-MSC-EXO and iPSC-MSC-EXO data sets. Differentially expressed miRNAs were identified through fold change > 1.5 and Q value < 0.001 with the threshold set for up- and down-regulated genes. Heat maps of differentially expressed miRNAs were generated by the omicshare cloud platform.

### Western blotting

Total protein of NMCMs with different treatments and mouse heart tissue from different groups was extracted using a total protein extraction kit following the protocol (Bestbio, BB-3101) and protein concentrations measured with a BCA assay kit (Thermo, 231227). 30 μg protein from different groups was resolved on SDS-PAGE gel and then transferred to PVDF membranes. After blocking in TBST with 5% fat-free milk, the PVDF membranes were incubated overnight at 4 °C with the following primary antibodies: anti-TSG101 (Abcam, ab125011), anti-Alix (Abcam, ab186429), anti-p-Drp1 (Ser616) (CST, 3455), anti-Drp1 (CST, 14647), anti-p21 (Abcam, ab109199), anti-p53 (Abcam, ab26), anti-Mfn1 (Abcam, ab57602), anti-Mfn2 (Abcam, ab124773), anti-VPO1 (FineTest, FNab10858), anti-p-ERK (CST, 9101), anti-ERK (CST, 4695), and anti-GAPDH (CST, 2118). Membranes were then washed three times with TBST and incubated at room temperature with secondary antibodies for 1 h. Finally, the membranes were exposed in a dark room and the density of protein bands quantified by Image J software (National Institutes of Health, Bethesda, MD, USA).

### Animal study

All animal procedures were approved by the Animal Research Committee of Guangdong Provincial People's Hospital (No.KY-Z-2022-053-02). A mouse model of DIC was established in ICR mice (6 ~ 8 weeks) by intraperitoneal injection of DOX (3 mg/kg each time, six times over two weeks with a total cumulative dose = 18 mg/kg) as described previously [24]. In the control group, mice were intraperitoneally injected with an equal volume of PBS. Three doses of BM-MSC-EXOs, iPSC-MSC-EXOs or miR-9-5p^KD^-iPSC-MSC-EXOs (30 μg), suspended in 100μL PBS, were injected through the tail vein of DIC mice on days 9, 11, and 13, respectively. Cardiac function was measured by transthoracic echocardiography (Ultramark 9; Soma Technology, Bloomfield, CT, United States) on days 0, 7, 14 and 35. The mice were anesthetized using 2% isoflurane and chest hair removed. Next, all mice were placed on a heating pad (37 °C). The mouse heart was imaged using M-mode via a two-dimensional parasternal long axis with heart rate ranging from 350–500 beats/min. Left ventricle fractional shortening (LVFS) and ejection fraction (LVEF) were calculated. To study the cardioprotective effect of miR-9-5p on DIC, another DIC model was established and three doses of miR-9-5p agomir (30 mg/kg) or the same dosage of control agomir were injected through the tail vein of DIC mice on days 9, 11, and 13. Cardiac function was measured by transthoracic echocardiography on day 0 and 35.

### Hematoxylin and eosin (H&E) staining

After heart function measurement on day 35, all mice were killed and heart tissue collected. Tissue was fixed, embedded, and cut into 5-μm sections. H&E staining was performed according to the manufacturer’s protocol (Servicebio, G1076). The percentage of cardiomyocyte vacuolization was calculated.

### Sirius red staining

After echocardiographic measurement on day 35, Sirius red staining was performed according to the protocol. Images from 6 mice for each group were captured. The percentage fibrotic area was determined as the total fibrotic area/the total LV area × 100%.

### TEM assay

The mitochondrial morphology in mouse heart tissue from different groups was examined by TEM assay as reported previously [15]. Images from 6 mice in each group were captured and at least 1000 mitochondria counted. Mitochondrial size was calculated using Image-Pro Plus software. Size < 0.6 μm^2^ was considered to indicate mitochondrial fragmentation.

### Statistical analysis

Data are expressed as mean ± SD. Statistical analyses were performed by GraphPad Prism 9.3.0. Comparison between two groups was assessed using unpaired Student’s t-test, and comparison among more than two groups by one-way-ANOVA followed by the Bonferroni test. A p value < 0.05 was considered statistically significant.

## Results

### Characterization of BM-MSC-EXOs and iPSC-MSC-EXOs

**Fig. 1:**
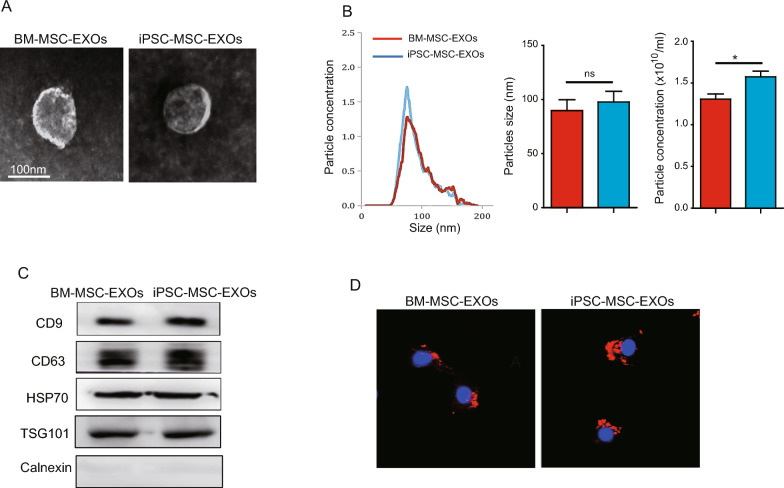
Characterization of BM-MSC-EXOs and iPSC-MSC-EXOs. **A** Representative TEM images showing cup-shaped morphology of BM-MSC-EXOs and iPSC-MSC-EXOs. **B** The particle concentration and size distribution of BM-MSC-EXOs and iPSC-MSC-EXOs were analyzed by NTA. **C** Representative images of Western blotting showing the exosomal protein markers including CD9, CD63, TSG101 and HSP70 in BM-MSC-EXOs and iPSC-MSC-EXOs. **D** Representative immunofluorescence images demonstrating the internalization of DiI-labeled BM-MSC-EXOs or iPSC-MSC-EXOs in NMCMs. n = 3. Data are expressed as mean ± SD. *p < *0.05*. ns = not significant

BM-MSC-EXOs and iPSC-MSC-EXOs were isolated and characterized by TEM, NTA and Western blotting. TEM revealed that both BM-MSC-EXOs and iPSC-MSC-EXOs exhibited a typical spheroid morphology with a double-layer membrane structure (Fig. 1A). NTA demonstrated that the particle diameters of BM-MSC-EXOs and iPSC-MSC-EXOs ranged from 30 to 150 nm (Fig. 1B). No difference in particle diameters was observed between BM-MSC-EXOs and iPSC-MSC-EXOs (Fig. 1B). Nevertheless compared with BM-MSC-EXOs, the concentration of particles was significantly enhanced in iPSC-MSC-EXOs (Fig. 1B). Western blotting results demonstrated that both BM-MSC-EXOs and iPSC-MSC-EXOs expressed specific exosomal molecular markers including CD9, CD63, TSG101 and HSP70 but not Calnexin (Fig. 1C). Next, to examine whether NMCMs could take up MSC-EXOs, DiI-labeled BM-MSC-EXOs and iPSC-MSC-EXOs were incubated with NMCMs under DOX challenge for 24 h. Confocal images showed that DiI-labeled MSC-EXOs were presented around the nucleus, indicating that NMCMs could uptake MSC-EXOs (Fig. 1D). Collectively, these data showed that both BM-MSC-EXOs and iPSC-MSC-EXOs were successfully isolated and these MSC-EXOs could be internalized by NMCMs.

### Transplantation of iPSC-MSC-EXOs improves cardiac function of DIC mice

**Fig. 2:**
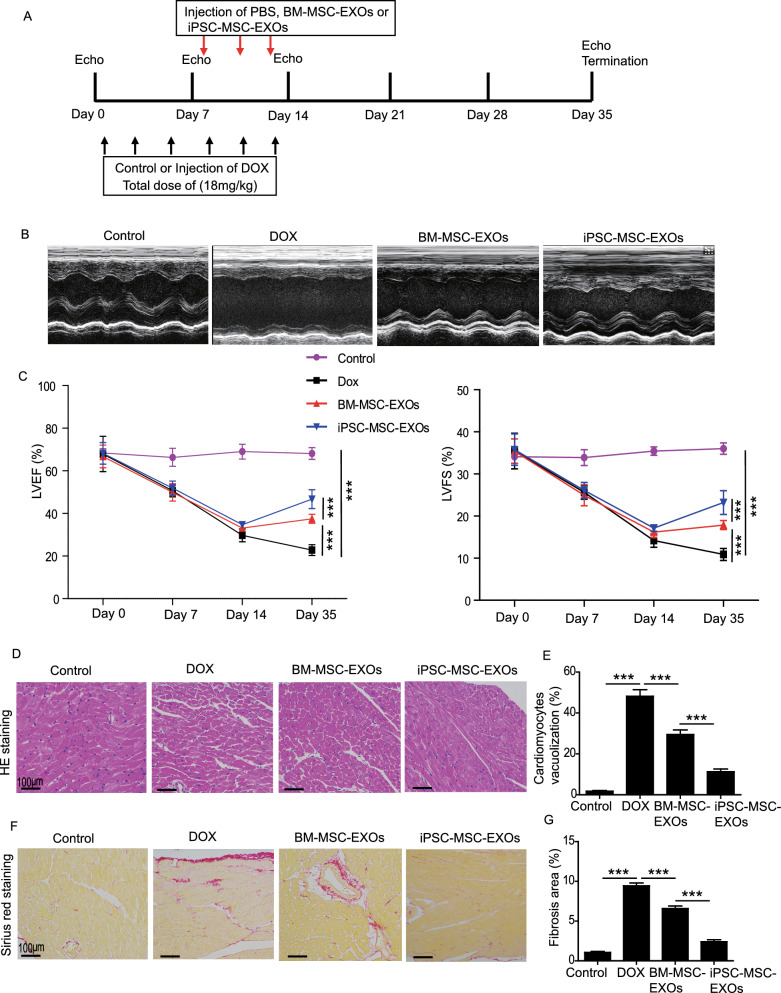
Administration of iPSC-MSC-EXOs attenuated DIC in mice. **A** Schematic chart showing the creation of a DIC model and administration of PBS, BM-MSC-EXOs or iPSC-MSC-EXOs. **B** Representative echocardiographic images were captured on day 35 after DOX treatment in mice treated with PBS, BM-MSC-EXOs or iPSC-MSC-EXOs or control mice. **C** The LVEF and LVFS were analyzed on Day 0, 7, 14 and 35 in controls or mice with DIC that received PBS, BM-MSC-EXO or iPSC-MSC-EXO treatment. **D** Representative images of HE staining showing myocardial histological changes in DIC mice treated with PBS, BM-MSC-EXOs and iPSC-MSC-EXOs or control mice. **E** Quantitative analysis of vacuolization in the heart tissue from DIC mice that received PBS, BM-MSC-EXOs or iPSC-MSC-EXOs treatment or control mice. **F** Representative images of Sirius red staining of heart sections from DIC mice that received PBS, BM-MSC-EXOs or iPSC-MSC-EXOs treatment and control mice. **G** Quantitative analysis of cardiac fibrosis in different experimental groups. Data are expressed as mean ± SD. n = 6 mice for each group, ***p < *0.001*

The protocol for animal experiments is outlined in Fig. 2A. To examine the cardioprotective effects of MSC-EXOs on DIC, 3 doses of BM-MSC-EXOs or iPSC-MSC-EXOs were injected into the tail vein of DIC mice on days 9, 11, and 13. Representative echocardiographic images on day 35 are shown in Fig. 2B. The mice heart rate from different groups was analyzed. There was no significant difference in heart rate between the control, MI, BM-MSC-EXO or iPSC-MSC-EXO groups (Additional file 1: Fig. S1). Compared with the control group, LVEF and LVFS were decreased over time up to day 35 in the DOX group, indicating that a mouse model of DIC had been established (Fig. 2C). Administration of both BM-MSC-EXOs and iPSC-MSC-EXOs greatly increased LVEF and LVFS on day 35, and injection of iPSC-MSC-EXOs further improved heart function in DIC mice compared with BM-MSC-EXOs (Fig. 2C). HE staining showed that administration of both BM-MSC-EXOs and iPSC-MSC-EXOs reduced DOX-induced extensive vacuolization in heart tissue, and iPSC-MSC-EXO treatment further decreased cardiomyocyte vacuolization (Fig. 2D, E). Cardiac fibrosis in different groups was examined by Sirius red staining (Fig. 2F). Compared with the control group, the ratio of cardiac fibrosis was greatly increased in the DOX group (Fig. 2G). Nevertheless treatment with both BM-MSC-EXOs and iPSC-MSC-EXOs significantly reduced cardiac fibrosis induced by DOX, and treatment with iPSC-MSC-EXOs further inhibited cardiac fibrosis in the heart of DIC mice (Fig. 2G). Taken together, these data demonstrated that transplantation of BM-MSC-EXOs and iPSC-MSC-EXOs significantly improved heart function and decreased cardiac fibrosis in DIC mice, and cardioprotection against DIC was superior with iPSC-MSC-EXOs.

### iPSC-MSC-EXOs ameliorate cardiomyocyte senescence and inhibit mitochondrial fragmentation in the heart of mice with DIC

**Fig. 3:**
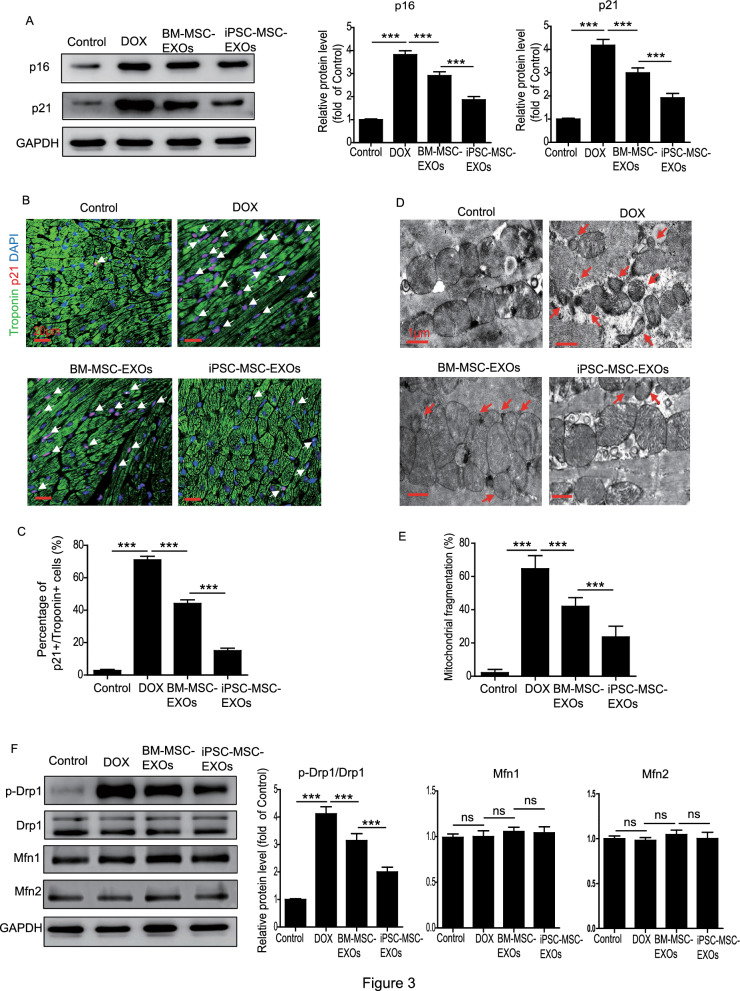
Administration of iPSC-MSC-EXOs inhibited mitochondrial fragmentation and cardiomyocyte senescence in hearts of DIC mice. **A** Western blotting and quantitative measurement of the protein level of p16 and p21 in DIC mice that received PBS, BM-MSC-EXOs or iPSC-MSC-EXOs treatment or control mice. **B** Representative images of Troponin and p21 double staining in the heart of DIC mice that received PBS, BM-MSC-EXOs or iPSC-MSC-EXOs treatment and control mice. **C** Quantitative measurement of Troponin^+^/p21^+^ double-positive cells in the heart of DIC mice that received PBS, BM-MSC-EXOs or iPSC-MSC-EXOs treatment and control mice. **D** Representative TEM images showing the mitochondria in the heart of DIC mice that received PBS, BM-MSC-EXOs or iPSC-MSC-EXOs treatment and control mice. **E** Quantitative measurement of mitochondrial fragmentation in the heart of DIC mice that received PBS, BM-MSC-EXOs or iPSC-MSC-EXOs treatment and control mice. **F** Western blotting and quantitative measurement of the protein level of p-Drp1/Drp1, Mfn1 and Mfn2 in the heart of DIC mice that received PBS, BM-MSC-EXOs or iPSC-MSC-EXOs treatment and control mice. Data are expressed as mean ± SD. n = 6 mice for each group, ***p < *0.001*, ns = not significant

Since DOX-induced cardiomyocyte senescence contributes to heart dysfunction, we determined whether the cardioprotective effects of iPSC-MSC-EXOs against DIC were achieved via regulation of cardiomyocyte senescence. Western blotting results showed that the expression of cellular senescence markers p16 and p21 was much lower in the BM-MSC-EXO and iPSC-MSC-EXO groups than the DOX group (Fig. 3A). Importantly, treatment with iPSC-MSC-EXOs further downregulated the expression of p16 and p21 compared with BM-MSC-EXO treatment (Fig. 3A). Next, we performed Troponin^+^/p21^+ ^double staining to evaluate cardiomyocyte senescence among the different groups (Fig. 3B). DOX treatment significantly increased the percentage of Troponin^+^/p21^+ ^-positive cells, indicating DOX-induced cardiomyocyte senescence (Fig. 3C). In contrast, the percentage of Troponin^+^/p21^+ ^-positive cells was greatly reduced following treatment with BM-MSC-EXOs and iPSC-MSC-EXOs, and further reduced with iPSC-MSC-EXO treatment (Fig. 3C). TEM analysis was performed to determine the mitochondrial dynamics and to quantify mitochondrial fragmentation in heart tissue from the different groups (Fig. 3D). Mitochondrial fragmentation was greatly enhanced in the heart tissue of mice with DOX treatment, and treatment with BM-MSC-EXOs and iPSC-MSC-EXOs significantly downregulated this fragmentation (Fig. 3E). Importantly, treatment with iPSC-MSC-EXOs was more efficient than BM-MSC-EXO treatment (Fig. 3E). Similarly, Western blotting showed that compared with the control group, the expression of mitochondrial fission protein p-Drp1/Drp1 was greatly upregulated in the DOX group but downregulated in the BM-MSC-EXO and iPSC-MSC-EXO group, to a greater extent in the latter (Fig. 3F). The mitochondrial fusion protein MFN1 and MFN2 was not significantly changed among groups (Fig. 3F). Taken together, these findings suggested that administration of iPSC-MSC-EXOs ameliorated mitochondrial fragmentation and cardiomyocyte senescence in the myocardial tissue of DOX-treated mice.

### iPSC-MSC-EXOs inhibit DOX-induced cardiomyocyte senescence via downregulation of mitochondrial fragmentation in vitro

**Fig. 4:**
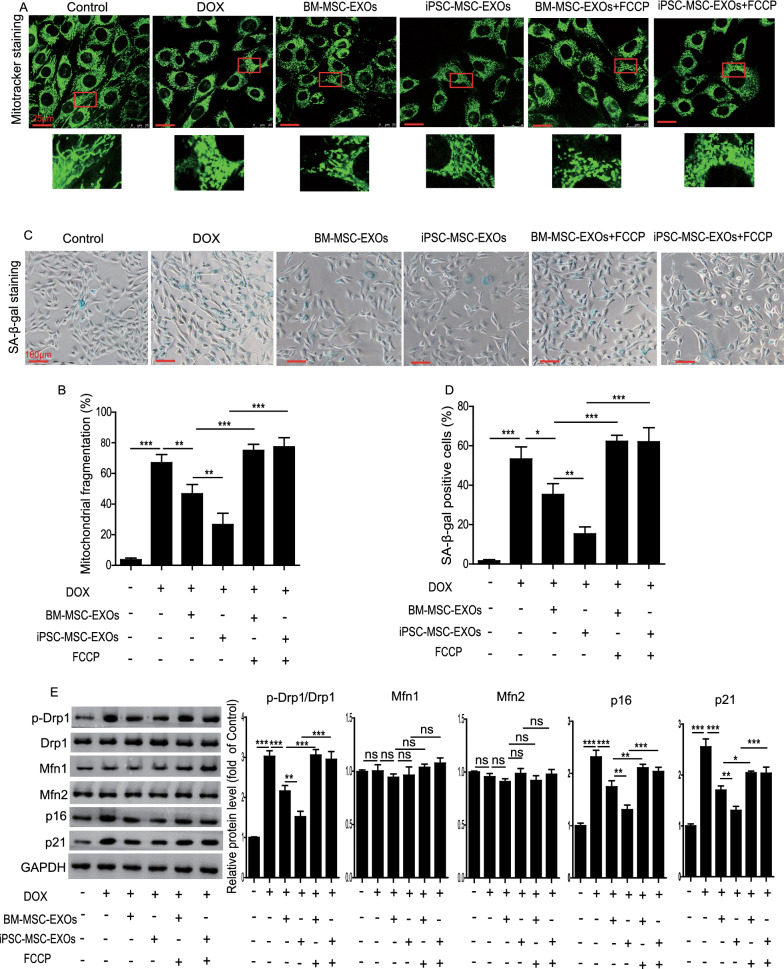
iPSC-MSC-EXOs ameliorated DOX-induced cardiomyocyte senescence via inhibiting mitochondrial fragmentation. **A** Representative images showing the fragmented mitochondria in control, DOX, DOX + BM-MSC-EXOs, DOX + iPSC-MSC-EXOs, DOX + BM-MSC-EXOs + FCCP, and DOX + iPSC-MSC-EXOs + FCCP-treated NMCMs. **B** Quantitative measurement of fragmented mitochondria in NMCMs from the different groups. **C** Representative SA-β-gal staining images in control, DOX, DOX + BM-MSC-EXOs, DOX + iPSC-MSC-EXOs, DOX + BM-MSC-EXOs + FCCP, and DOX + iPSC-MSC-EXOs + FCCP-treated NMCMs. **D** Quantitative measurement of SA-β-gal positive NMCMs from the different groups. **E** Western blotting and quantitative measurement of the protein level of p-Drp1/Drp1, Mfn1/2, p16 and p21 in control, DOX, DOX + BM-MSC-EXOs, DOX + iPSC-MSC-EXOs, DOX + BM-MSC-EXOs + FCCP, and DOX + iPSC-MSC-EXOs + FCCP-treated NMCMs. n = 3 biological replicates for each group. Data are expressed as mean ± SD. *p < 0.05, **p < 0.01, ***p < *0.001*, ns = not significant

We further determined whether iPSC-MSC-EXOs could inhibit DOX-induced cardiomyocyte senescence by ameliorating mitochondrial fragmentation in vitro. As shown in Figure S2, NMCM senescence was induced by DOX in a time-dependent manner (Additional file 1: Fig. S2). Next, we treated NMCMs with BM-MSC-EXOs or iPSC-MSC-EXOs under DOX challenge for 72 h. DOX treatment significantly increased mitochondrial fragmentation (Fig. 4A, B) and enhanced SA-β-gal activity (Fig. 4C, D) in NMCMs. Western blotting also revealed that DOX treatment increased the protein level of p-Drp1/Drp, p21 and p16 in NMCMs (Fig. 4E). BM-MSC-EXO and iPSC-MSC-EXO treatment greatly inhibited mitochondrial fragmentation (Fig. 4A, B) and SA-β-gal activity (Fig. 4C, D), and downregulated the protein level of p-Drp1/Drp1, p21 and p16 (Fig. 4E) in DOX-induced NMCMs. Notably, iPSC-MSC-EXOs were superior to BM-MSC-EXOs in attenuation of DOX-induced NMCM senescence and mitochondrial fragmentation (Fig. 4A–E). Nevertheless the suppressive effects of BM-MSC-EXOs and iPSC-MSC-EXOs on DOX-induced NMCM mitochondrial fragmentation and senescence were partially reversed by FCCP, a mitochondrial fission activator (Fig. 4A–E). Collectively, these results showed that iPSC-MSC-EXOs inhibited DOX-induced cardiomyocyte senescence via amelioration of mitochondrial fragmentation.

### miR-9-5p was the candidate effector of iPSC-MSC-EXOs in attenuation of DOX-induced cardiomyocyte senescence

**Fig. 5:**
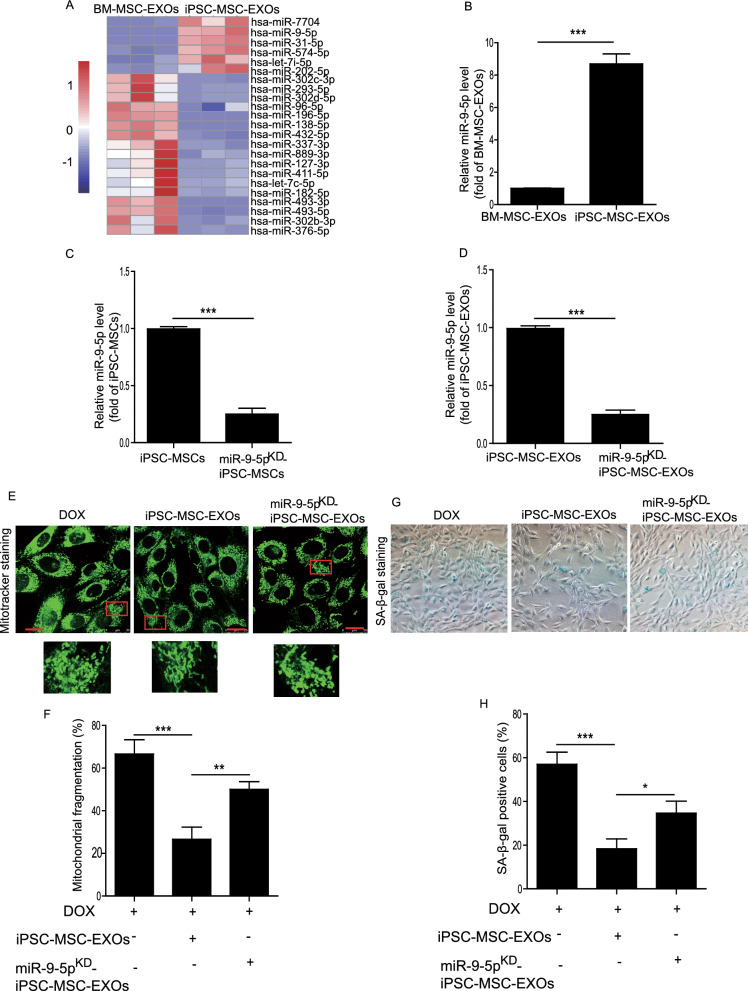
miR-9-5p was the candidate effector of iPSC-MSC-EXOs in attenuation of DOX-induced cardiomyocyte senescence. **A** RNA-seq analysis revealed the differential expression of miRNAs between BM-MSC-EXOs and iPSC-MSC-EXOs. **B** The expression level of miR-9-5p in iPSC-MSC-EXOs was validated by qPCR. **C** The expression level of miR-9-5p in iPSC-MSCs and miR-9-5p^KD^- iPSC-MSCs was validated by qPCR. **D** The expression level of miR-9-5p in iPSC-MSC-EXOs and miR-9-5p^KD^-iPSC-MSC-EXOs was validated by qPCR. **E**, **F** Representative images and quantitative measurement of mitochondrial fragmentation in NMCM treated with iPSC-MSC-EXOs or miR-9-5p^KD^-iPSC-MSC-EXOs under DOX challenge. **G**, **H** Representative images and quantitative measurement of SA-β-gal positive cells in NMCM treated with iPSC-MSC-EXOs or miR-9-5p^KD^-iPSC-MSC-EXOs under DOX challenge. n = 3 biological replicates for each group. Data are expressed as mean ± SD. *p < 0.05, **p < 0.01, ***p < *0.001*

To determine how iPSC-MSC-EXOs inhibited DOX-induced mitochondrial fragmentation and senescence in cardiomyocytes, miRNA–seq was performed to identify the potential candidate miRNA between BM-MSC-EXOs and iPSC-MSC-EXOs. The miRNAs differentially expressed in BM-MSC-EXOs and iPSC-MSC-EXOs are shown in Fig. 5A. We focused mainly on the increased expression of miRNAs in iPSC-MSC-EXOs. Previous studies have reported that miR-9-5p plays a critical role in regulating cardiovascular diseases [25, 26]. Indeed, qPCR results confirmed that the level of miR-9-5p was significantly enhanced in iPSC-MSC-EXOs compared with BM-MSC-EXOs (Fig. 5B). Hence, we determined the effects of exosomal miR-9-5p in iPSC-MSC-EXOs on DOX-induced NMCM senescence in subsequent experiments. We first used miR-9-5p inhibitor to treat iPSC-MSCs and then isolated the EXOs. Results from qPCR demonstrated that miR-9-5p inhibitor treatment remarkably downregulated miR-9-5p content of iPSC-MSCs (Fig. 5C). More importantly, the level of miR-9-5p was greatly decreased in EXOs derived from miR-9-5p inhibitor-treated iPSC-MSCs (miR-9-5p^KD^-iPSC-MSC-EXOs) compared with iPSC-MSC-EXOs (Fig. 5D). Next, we assessed the effects of miR-9-5p^KD^-iPSC-MSC-EXOs on DOX-induced NMCM mitochondrial fragmentation and senescence. As shown in Fig. 5E, compared with the DOX group, mitochondrial fragmentation of NMCMs was greatly reduced in iPSC-MSC-EXOs but increased in miR-9-5p^KD^-iPSC-MSC-EXOs (Fig. 5E, F). Similar results were observed for SA-β-gal assay. Compared with iPSC-MSC-EXOs, administration of miR-9-5p^KD^-iPSC-MSC-EXOs failed to prevent DOX-induced NMCM senescence (Fig. 5G, H). To verify the effects of miR-9-5p on DOX-induced NMCM injury, we treated NMCMs with miR-9-5p mimic under DOX challenge for 72 h. MiR-9-5p mimic treatment significantly inhibited DOX-induced cellular senescence (Additional file 1: Fig. S3A, B) and mitochondrial fragmentation in NMCMs (Additional file 1: Fig. S3C, D). To further determine the cardioprotective effects of miR-9-5p on DIC, 3 doses of miR-9-5p agomir were transplanted into the tail vein of DIC mice on days 9, 11, and 13 (Additional file 1: Fig. S4A). MiR-9-5p agomir treatment greatly improved heart function as evidenced by increased LVEF and LVFS (Additional file 1: Fig. S4B, C) and reduced cardiac fibrosis compared with DOX group (Additional file 1: Fig. S4D, E). Furthermore, agomir miR-9-5p treatment ameliorated cardiomyocyte senescence (Additional file 1: Fig. S4F, G) and downregulated mitochondrial fragmentation as shown by the decreased protein level of p-Drp1/Drp in heart tissue from DOX-treated mice (Additional file 1:Fig. S4H). Overall, these results revealed that miR-9-5p was the key component of iPSC-MSC-EXOs in attenuation of DOX-induced cardiomyocyte senescence.

### Exosomal miR-9-5p in iPSC-MSC-EXOs inhibited cardiomyocyte mitochondrial fission by inhibiting the VPO1/ERK signaling pathway

**Fig. 6:**
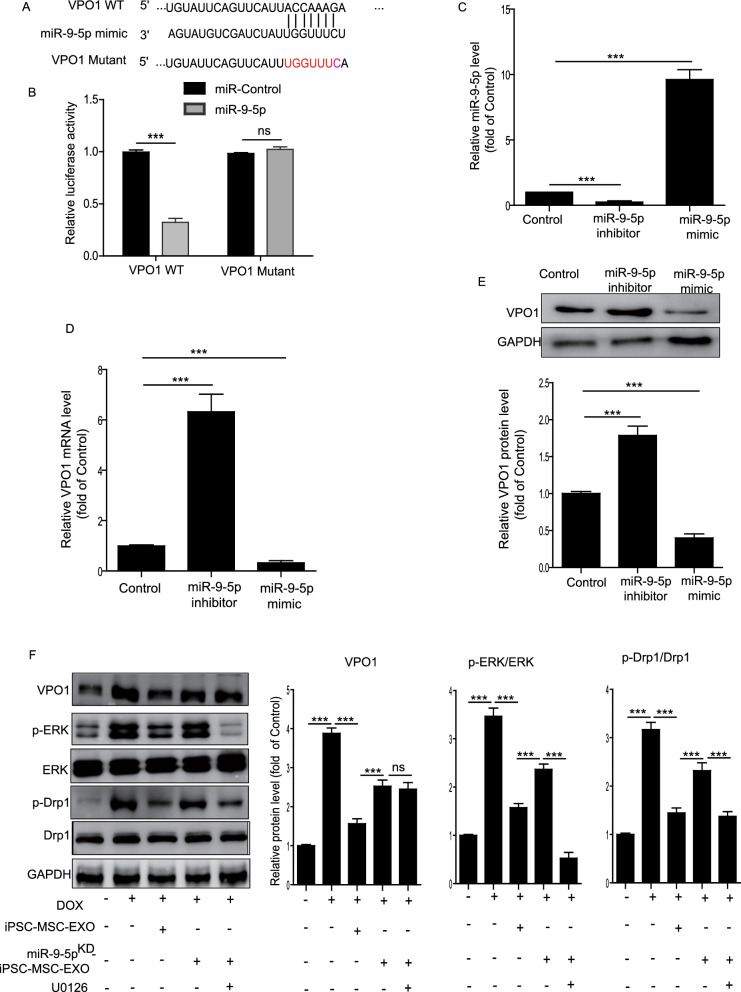
Exosomal miR-9-5p in iPSC-MSC-EXOs inhibited cardiomyocyte mitochondrial fission by inhibiting VPO1/ERK signaling pathway. **A** Bioinformatics analysis predicted the binding sites between VPO1 and miR-9-5p. **B** Luciferase reporter assays confirmed the association of VPO1 and miR-9-5p. **C** qRT-PCR analysis of the level of miR-9-5p in NMCMs treated with control, miR-9-5p mimic or miR-9-5p inhibitor. **D** qRT-PCR analysis of the level of VPO1 in NMCMs treated with control, miR-9-5p mimic or miR-9-5p inhibitor. **E** Western blotting analysis of the level of VPO1 in NMCMs treated with control, miR-9-5p mimic or miR-9-5p inhibitor. **F** Western blotting analysis of the level of VPO1, p-Drp1, Drp1, p-ERK, ERK in control, DOX, DOX + iPSC-MSC-EXOs, DOX + miR-9-5p^KD^-iPSC-MSC-EXOs and DOX + miR-9-5p^KD^-iPSC-MSC-EXOs + U0126 treated NMCMs. n = 3 biological replicates for each group. Data are expressed as mean ± SD.  ***p < *0.001,* ns = not significant

Gene prediction tools revealed that vascular peroxidase 1 (VPO1), also known as Peroxidasin (PXDN), is a potential target for miR‑9-5p (Fig. 6A). The relationship between VPO1 and miR‑9-5p was verified by luciferase reporter assays (Fig. 6B). We used miR-9-5p inhibitor or mimic to treat NMCMs, and found that the level of miR-9-5p was upregulated in miR-9-5p mimic-treated NMCMs but downregulated in miR-9-5p inhibitor-treated NMCMs (Fig. 6C). Furthermore, the mRNA and protein level of VPO1 were significantly reduced in miR-9-5p mimic-treated NMCMs but increased in miR-9-5p inhibited-treated NMCMs (Fig. 6D, E). Previous studies have reported that VPO1 plays a vital role in cardiovascular disease by regulating the ERK1/2 pathway [27, 28]. These results led us to determine whether the VPO1/ERK signaling pathway mediated DIC via regulation of mitochondrial dynamics. We first determined the protein level of VPO1 and p-ERK in DIC mice treated with BM-MSC-EXOs or iPSC-MSC-EXOs. Compared with the control group, the expression of VPO1 and p-ERK was greatly upregulated in the DOX group (Additional file 1: Fig. S5). Nevertheless injection of both BM-MSC-EXOs and iPSC-MSC-EXOs significantly reduced the expression of VPO1 and p-ERK in the heart tissue of DIC mice, iPSC-MSC-EXOs to a greater extent (Additional file 1: Fig. S5). To further verify that exosomal miR-9-5p in iPSC-MSC-EXOs inhibits cardiomyocyte mitochondrial fragmentation via inhibition of the VPO1/ERK signaling pathway, we treated NMCMs with miR-9-5p^KD^-iPSC-MSC-EXOs under DOX challenge. We found that iPSC-MSC-EXO treatment dramatically inhibited the expression of VPO1, p-ERK/ERK and p-Drp1/Drp1 in DOX-treated NMCMs (Fig. 6F). Nonetheless the expression of VPO1, p-ERK/ERK and p-Drp1/Drp1 was much higher in the miR-9-5p^KD^-iPSC-MSC-EXO group than the iPSC-MSC-EXO group. These effects were largely reversed by ERK inhibitor U0126 (Fig. 6F). These data demonstrate that exosomal miR-9-5p in iPSC-MSC-EXOs inhibited cardiomyocyte mitochondrial fission by inhibiting the VPO1/ERK signaling pathway.

### Knockdown of miR-9-5p reduced the cardioprotective effects of iPSC-MSC-EXOs in DIC

**Fig. 7:**
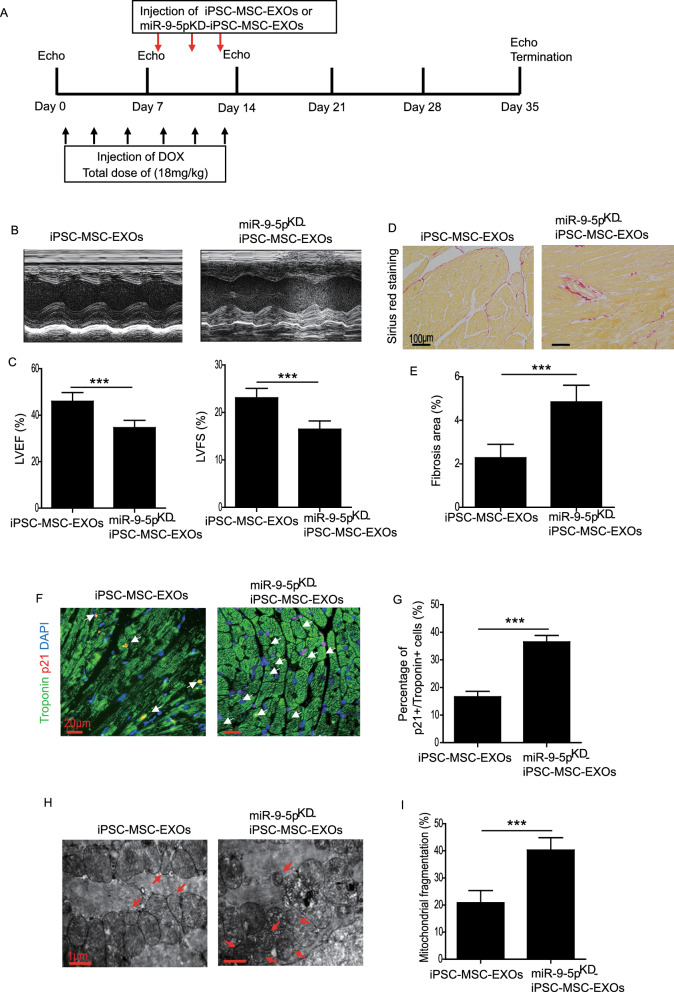
Knockdown of miR-9-5p impaired the cardioprotection afforded by iPSC-MSC-EXOs against DIC. **A** Schematic chart showing the introduction of DIC model and administration of iPSC-MSC-EXOs or miR-9-5p^KD^-iPSC-MSC-EXOs. **B** Representative echocardiographic images were captured on day 35 after DOX treatment in mice that received iPSC-MSC-EXOs or miR-9-5p^KD^-iPSC-MSC-EXOs. **C** The LVEF and LVFS were analyzed at 35 days in DIC mice that received iPSC-MSC-EXO or miR-9-5p^KD^-iPSC-MSC-EXO treatment. **D** Representative images of Sirius red staining of heart sections from DIC mice that received iPSC-MSC-EXO or miR-9-5p^KD^-iPSC-MSC-EXO treatment. **E** Quantitative analysis of cardiac fibrosis in DIC mice that received iPSC-MSC-EXO or miR-9-5p^KD^-iPSC-MSC-EXO treatment. **F** Representative images of Troponin and p21 double staining in the heart of DIC mice that received iPSC-MSC-EXO or miR-9-5p^KD^-iPSC-MSC-EXO treatment. **G** Quantitative measurement of Troponin^+^/p21^+^ double-positive cells in the heart of DIC mice that received iPSC-MSC-EXO or miR-9-5p^KD^-iPSC-MSC-EXO treatment. **H** Representative TEM images showing the mitochondria in the heart of DIC mice that received iPSC-MSC-EXO or miR-9-5p^KD^-iPSC-MSC-EXO treatment. **I** Quantitative measurement of mitochondrial fragmentation in the heart of DIC mice that received iPSC-MSC-EXO or miR-9-5p^KD^-iPSC-MSC-EXO treatment. Data are expressed as mean ± SD. n = 6 mice for each group, ***p < *0.001*

To further verify the cardioprotection afforded by exosomal miR-9-5p in iPSC-MSC-EXOs against DIC, we administrated miR-9-5p^KD^-iPSC-MSC-EXOs in a mouse model of DIC. The experimental protocol is outlined in Fig. 7A. The heart function of DIC mice that received miR-9-5p^KD^-iPSC-MSC-EXO treatment was evaluated. Compared with the iPSC-MSC-EXO group, LVEF and LVFS were remarkably decreased in the miR-9-5p^KD^-iPSC-MSC-EXO group, suggesting the reduced cardioprotective effects of miR-9-5p^KD^-iPSC-MSC-EXOs on DIC mice (Fig. 7B, C). Sirius red staining demonstrated a markedly increased level of myocardial fibrosis in the miR-9-5p^KD^-iPSC-MSC-EXO group compared with the iPSC-MSC-EXO group (Fig. 7D, E). The percentage of senescent cardiomyocytes as evidenced by Troponin^+^/p21^+^ double-positive cells was significantly enhanced in the miR-9-5p^KD^-iPSC-MSC-EXO group compared with the iPSC-MSC-EXO group (Fig. 7F, G). Diminishing miR-9-5p greatly impaired the regulatory effect of iPSC-MSC-EXOs on mitochondrial fragmentation induced by DOX in heart tissue (Fig. 7H, I). Collectively, these results reveal that loss of miR-9-5p weakened the cardioprotective effect of iPSC-MSC-EXOs against DIC, suggesting that miR-9-5p present in iPSC-MSC-EXOs plays a critical role in restoring heart function in a mouse model of DIC.

## Discussion

This study generated several major findings. First, DOX treatment induced cardiomyopathy by stimulating cardiomyocyte senescence via activation of mitochondrial fragmentation. Second, iPSC-MSC-EXOs were superior to BM-MSC-EXOs in attenuating DIC via amelioration of cardiomyocyte senescence. Third, compared with BM-MSC-EXOs, the enhanced efficacy of iPSC-MSC-EXOs in treating DIC could be attributed to their elevated level of miR-9-5p that is transferred into cardiomyocytes to inhibit mitochondrial fragmentation by regulating the VPO1/ERK signaling pathway. Finally, knockdown of miR-9-5 partially abrogated the cardioprotective effects of iPSC-MSC-EXOs in DIC.

There is strong evidence that EXOs derived from adult MSCs exert convincing therapeutic efficacy in cardiovascular diseases including DIC [29, 30]. Transplantation of BM-MSC-EXOs has been shown to functionally alleviate DIC in mice via suppression of the inflammatory response of cardiomyocytes and inflammation-related cell death [31]. In vitro study has also revealed that BM-MSC-EXO treatment robustly restrained DOX-induced pyroptosis and oxidative stress of myocardial cells via diminished GSDMD expression by regulating the PI3K-AKT-Foxo1 pathway [32]. To obtain a large volume of MSC-EXOs for transplantation, BM-MSCs need to be expanded extensively in vitro. Nevertheless BM-MSCs easily become senescent after long-term culture, therefore impairing the quantity and quality of MSC-EXOs. Thus, exploring an alternative type of adult MSCs is vital. We and our collaborators have reported that iPSC-MSCs exhibit a superior therapeutic effect in terms of cardiovascular repair to BM-MSCs due to their superior paracrine actions, making them an ideal strategy for DIC therapy [23, 33]. Nonetheless the therapeutic efficacy of iPSC-MSC-EXOs in DIC has not been determined. In the current study, transplantation of BM-MSC-EXOs and iPSC-MSC-EXOs significantly improved heart function and decreased cardiac fibrosis in a mouse model of DIC. In addition, iPSC-MSC-EXOs exhibited superior cardioprotective effects in DIC. Recently, many studies have indicated that in addition to excessive reactive oxygen species generation, apoptosis and pyroptosis, cardiomyocyte senescence plays an essential role in regulating DIC [34, 35]. DOX induced cardiomyocyte senescence via activation of p38 MAPK/Redd1/NF-κB, leading to heart dysfunction in mice [36]. Our study confirmed that DOX induced the senescent phenotype of cardiomyocytes. More importantly, consistent with our previous study [15], we found in the present study that DOX induced cardiomyocyte senescence via activation of mitochondrial fragmentation. In the in vivo study, systemic BM-MSC-EXO and iPSC-MSC-EXO administration prevented DOX-induced cardiomyocyte mitochondrial fragmentation and senescence. Furthermore, iPSC-MSC-EXOs were superior to BM-MSC-EXOs and these effects were partially abrogated by mitochondrial fission activator, FCCP. Therefore, our results showed that transplantation of iPSC-MSC-EXOs improved cardiac function in DIC mice via amelioration of mitochondrial fragmentation-mediated cardiomyocyte senescence.

miRNAs enriched in MSC-EXOs are the key biological components that promote repair of myocardial injury in cardiovascular disease [37, 38]. It has been reported that BM-MSC-EXOs protect the myocardium against DOX-induced toxicity at least partially by delivering miR-96 via inhibition of the Rac/NF-κB signaling pathway [39]. Lee et al. revealed that administration of MSC-derived small extracellular vesicles attenuated DIC by increasing survivin expression through the delivery of miR-199a-3p [18]. To identify the key candidate miRNA in iPSC-MSC-EXOs that exert a sustained cardioprotective effect in DIC, we performed miRNA–seq and analyzed the miRNAs differentially expressed between BM-MSC-EXOs and iPSC-MSC-EXOs. We focused on miR-9-5p for several major reasons. First, miR-9-5p was the most highly enriched miRNA in iPSC-MSC-EXOs vs BM-MSC-EXOs. Second, although the expression of miR-9-5p is closely associated with cardiovascular diseases [40], the cardiovascular function of miR-9-5p is unclear. Third, bioinformatic analysis and quantitative assessment confirmed that miR-9-5p directly targets VPO1 in cardiomyocytes, as validated by luciferase reporter assays. It has been well established that VPO1 plays an important role in regulating myocardial damage [41]. The expression of VPO1 was greatly increased in the mouse heart following ischemia/reperfusion (I/R) injury, and knockdown of VPO1 exerted a beneficial effect on I/R injury [42]. More importantly, VPO1 has been identified as an ERK1/2 pathway activator [43, 44]. Meanwhile, ERK1/2 activation has been shown to induce mitochondrial fragmentation [45, 46]. Nonetheless the role of the VPO1/ERK1/2 signal pathway in DIC has not been determined. In the current study, the expression of VPO1 and p-ERK1/2 was significantly upregulated in the heart tissue of a mouse DIC model and DOX-treated NMCMs. We also found that both BM-MSC-EXO and iPSC-MSC-EXO treatment significantly inhibited DOX-induced cardiomyocyte mitochondrial fragmentation and senescence, concomitant with suppressed VPO1 and p-ERK1/2 expression. iPSC-MSC-EXO treatment further decreased the VPO1 and p-ERK1/2 expression in the DIC hearts and was superior to treatment with BM-MSC-EXOs. Knockdown of miR-9-5p in iPSC-MSC-EXOs blunted the cardioprotection against DIC, as well as increased VPO1 and p-ERK1/2 expression, indicating that iPSC-MSC-exosomal miR-9-5p elicits cardioprotective effects partly via inhibition of the VPO1/ERK signal pathway in DIC hearts. Importantly, we also found that the expression of VPO1 and p-ERK/ERK after BM-MSC-EXO treatment was significantly reduced even they are not miR-9-5p enriched, indicating that some other molecular substances in BM-MSC-EXOs protect against DIC via direct or indirect targeting of the VPO1/ERK signal pathway.

There are some limitations in the current study that should be highlighted. First, in addition to miR-9-5p, the function of other molecular substances that are enriched in iPSC-MSC-EXOs on DIC remain to be investigated. Second, it is unclear whether iPSC-MSC-EXOs mediate other targets including telomere shortening or autophagy dysfunction to inhibit cardiomyocyte senescence in DIC. Third, besides the VPO1/ERK signaling pathway, further research is needed to ascertain whether miR-9-5p regulates other downstream pathways to effectively inhibit mitochondrial fission. Fourth, the protective effects of iPSC-MSC-EXOs on endothelial cells or fibroblast injury in DIC should be determined. Finally, in the current study, we investigated only the cardioprotective effects of iPSC-MSC-EXOs on DIC in a cellular and animal model. Despite this, our results shed new light on the clinical application of iPSC-MSC-EXOs as a novel therapeutic strategy for DIC treatment. Further clinical studies are warranted to validate our findings.

## Conclusion

Our study shows that iPSC-MSC-EXOs are more effective than BM-MSCs-EXOs for cardioprotection against DIC, primarily due to their abundant miR-9-5p, a crucial molecular component. iPSC-MSC-EXO-derived exosomal miR-9-5p protects the myocardium against DIC by ameliorating mitochondrial fission-mediated cardiomyocyte senescence via regulation of the VPO1/ERK signal pathway. The present study highlights that iPSC-MSC-EXOs can serve as a novel therapeutic strategy for DIC treatment.

### Supplementary Information

**Additional file 1: Figure S1.** The heart rate of control or mice with DOX that received PBS, BM-MSC-EXO or iPSC-MSC-EXO treatment. Data are expressed as mean±SD. n = 6 mice for each group. ns=not significant. **Figure S2.** DOX induced NMCM senescence in a time-dependent manner. Representative images of SA-β-gal staining in NMCMs and quantitative of SA-β-gal positive NMCMs under DOX treatment for 0h, 24h, 48h, 72h and 96h. n = 3 biological replicates for each group. Data are expressed as mean±SD, ***p<0.001. ns=not significant. **Figure S3.** MiR-9-5p mimic treatment inhibited DOX-induced NMCM mitochondrial fragmentation and senescence (A) Representative SA-β-gal staining images in control, DOX, DOX+miR-9-5p mimic-treated NMCMs. (B) Quantitative measurement of SA-β-gal positive NMCMs from the different groups. (C) Representative images showing the fragmented mitochondria in control, DOX, DOX+miR-9-5p mimic-treated NMCMs. (D) Quantitative measurement of fragmented mitochondria in NMCMs from the different groups. n = 3 biological replicates for each group. Data are expressed as mean ± SD. **p < 0.01, ***p < 0.001. **Figure S4.** Administration of miR-9-5p agomir improved heart function and inhibited mitochondrial fragmentation and cardiomyocyte senescence in hearts of DIC mice. (A) Schematic chart showing the creation of a DIC model and administration of control agomir, or miR-9-5p agomir. (B) Representative echocardiographic images were captured on day 35 after DOX treatment in mice treated with control agomir or miR-9-5p agomir. (C) The LVEF and LVFS were analyzed on Day 0 and 35 in controls or mice with DIC that received control agomir or miR-9-5p agomir treatment. (D) Representative images of Sirius red staining of heart sections from DIC mice that received control agomir or miR-9-5p agomir and control mice. (E) Quantitative analysis of cardiac fibrosis in different experimental groups. (F) Representative images of Troponin and p21 double staining in the heart of DIC mice that received control agomir or miR-9-5p agomir and control mice. (G) Quantitative measurement of Troponin+/p21+ double-positive cells in the heart of DIC mice that received control agomir or miR-9-5p agomir and control mice. (H) Western blotting and quantitative measurement of the protein level of p-Drp1/Drp1, Mfn1 and Mfn2 in the heart of DIC mice that received control agomir or miR-9-5p agomir treatment and control mice. Data are expressed as mean ± SD. n = 6 mice for each group, ***p < 0.001, ns = not significant. **Figure S5.** Western blotting and quantitative analysis of the expression level of VPO1 and p-ERK in heart tissues from control or mice with DOX that received PBS, BM-MSC-EXO or iPSC-MSC-EXO treatment. Data are expressed as mean±SD. n = 6 mice for each group, ***p<0.001. **Figure S6.** Original Western blotting images.

## Data Availability

Data will be made available on request.
