# Supplementary material for: Exosomal miR-9-5p derived from iPSC-MSCs ameliorates doxorubicin-induced cardiomyopathy by inhibiting cardiomyocyte senescence
Source: J Nanobiotechnology. 2024 Apr 20;22:195. doi: 10.1186/s12951-024-02421-8 (PMC11032595; doi:10.1186/s12951-024-02421-8)
Supplement: Supplementary file 1 — Additional file 1: Figure S1. The heart rate of control or mice with DOX that received PBS, BM-MSC-EXO or iPSC-MSC-EXO treatment. Data are expressed as mean±SD. n = 6 mice for each group. ns=not significant. Figure S2. DOX induced NMCM senescence in a time-dependent manner. Representative images of SA-β-gal staining in NMCMs and quantitative of SA-β-gal positive NMCMs under DOX treatment for 0h, 24h, 48h, 72h and 96h. n = 3 biological replicates for each group. Data are expressed as mean±SD, ***p<0.001. ns=not significant. Figure S3. MiR-9-5p mimic treatment inhibited DOX-induced NMCM mitochondrial fragmentation and senescence (A) Representative SA-β-gal staining images in control, DOX, DOX+miR-9-5p mimic-treated NMCMs. (B) Quantitative measurement of SA-β-gal positive NMCMs from the different groups. (C) Representative images showing the fragmented mitochondria in control, DOX, DOX+miR-9-5p mimic-treated NMCMs. (D) Quantitative measurement of fragmented mitochondria in NMCMs from the different groups. n = 3 biological replicates for each group. Data are expressed as mean ± SD. **p < 0.01, ***p < 0.001. Figure S4. Administration of miR-9-5p agomir improved heart function and inhibited mitochondrial fragmentation and cardiomyocyte senescence in hearts of DIC mice. (A) Schematic chart showing the creation of a DIC model and administration of control agomir, or miR-9-5p agomir. (B) Representative echocardiographic images were captured on day 35 after DOX treatment in mice treated with control agomir or miR-9-5p agomir. (C) The LVEF and LVFS were analyzed on Day 0 and 35 in controls or mice with DIC that received control agomir or miR-9-5p agomir treatment. (D) Representative images of Sirius red staining of heart sections from DIC mice that received control agomir or miR-9-5p agomir and control mice. (E) Quantitative analysis of cardiac fibrosis in different experimental groups. (F) Representative images of Troponin and p21 double staining in the heart of DIC mice that [file 12951_2024_2421_MOESM1_ESM.pdf]

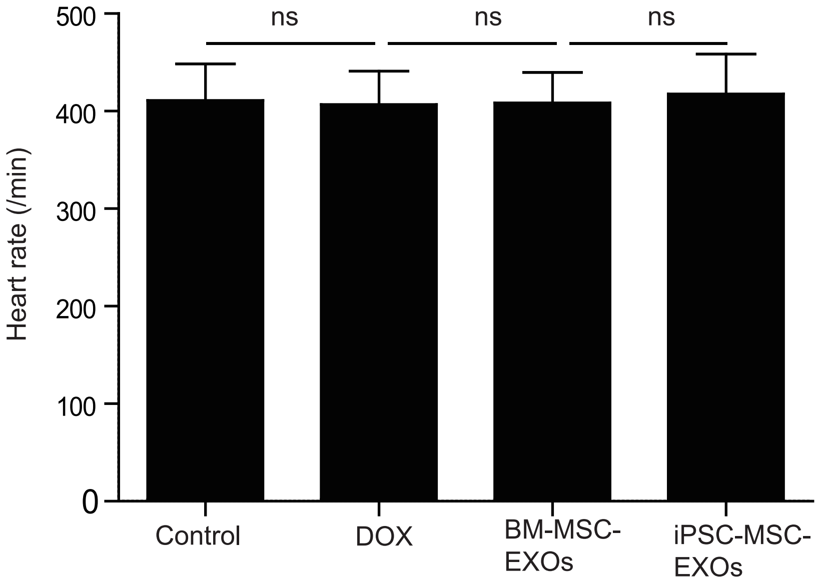

Heart rate (/min)
500
400
300
200
100
0
ns
ns
ns
Control
DOX
BM-MSC-
EXOs
iPSC-MSC-
EXOs

Figure S1

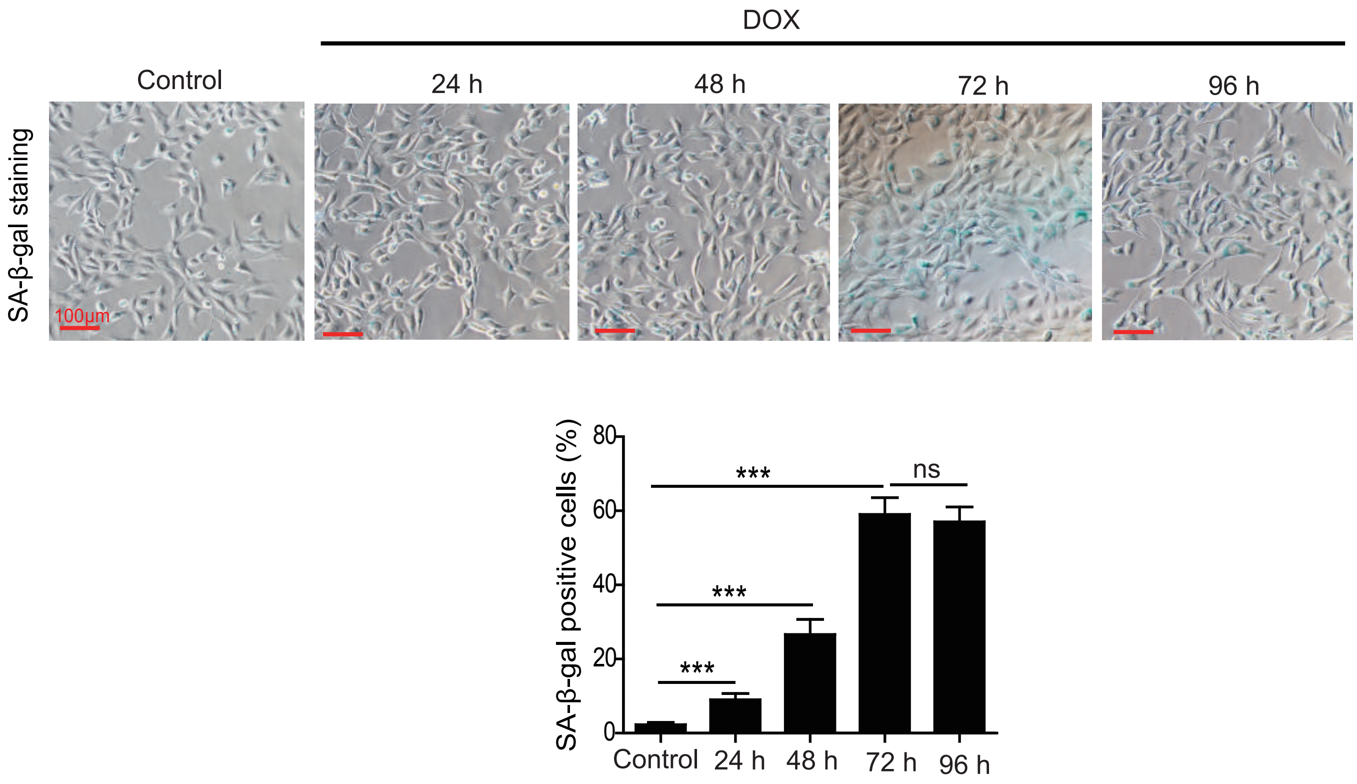
DOX
Control
24 h
48 h
72 h
96 h
SA-β-gal staining
100μm
SA-β-gal positive cells (%)
80
60
40
20
0
***
***
***
ns
Control
24 h
48 h
72 h
96 h

Figure S2

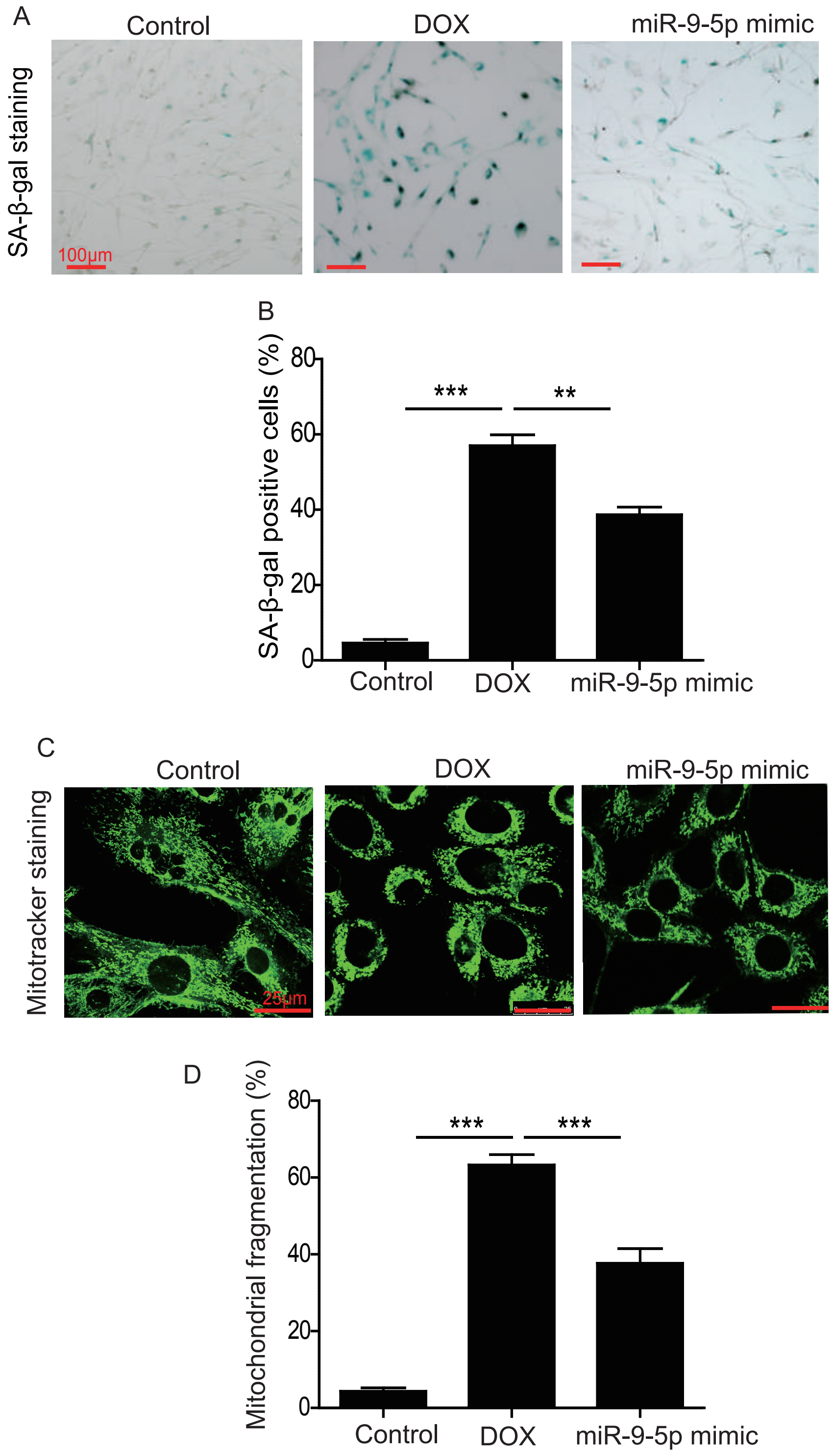
A
Control
DOX
miR-9-5p mimic
SA-β-gal staining
100μm
B
SA-β-gal positive cells (%)
80
60
40
20
0
***
**
Control
DOX
miR-9-5p mimic
C
Control
DOX
miR-9-5p mimic
Mitotracker staining
25μm
D
Mitochondrial fragmentation (%)
80
60
40
20
0
***
***
Control
DOX
miR-9-5p mimic

Figure S3

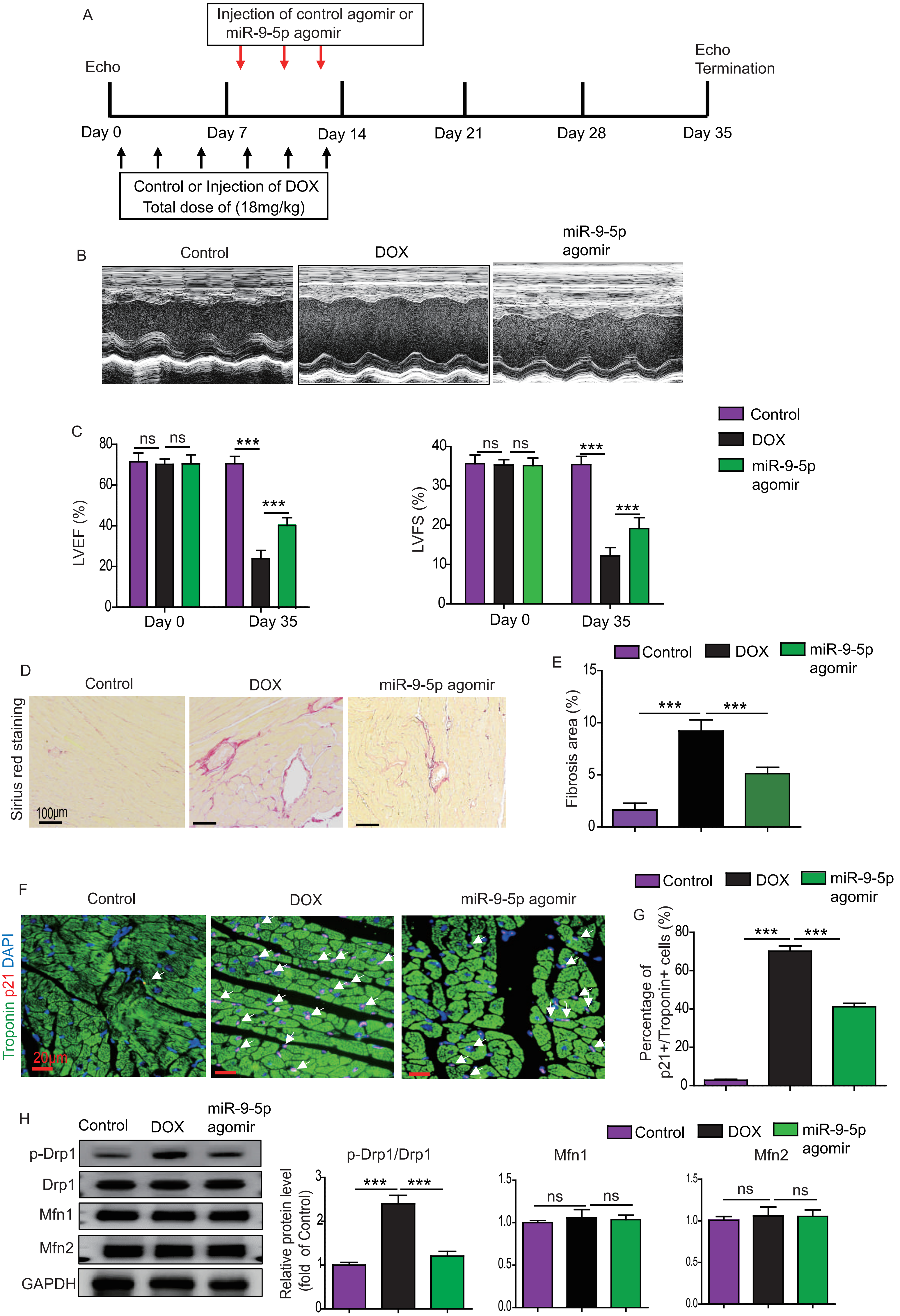

A
Injection of control agomir or miR-9-5p agomir
Echo
Echo Termination
Day 0
Day 7
Day 14
Day 21
Day 28
Day 35
Control or Injection of DOX Total dose of (18mg/kg)
B
Control
DOX
miR-9-5p agomir
C
LVEF (%)
LVFS (%)
ns
***
Day 0
Day 35
Control
DOX
miR-9-5p agomir
D
Sirius red staining
100μm
E
Fibrosis area (%)
F
Troponin p21 DAPI
20μm
G
Percentage of p21+/Troponin+ cells (%)
H
p-Drp1
Drp1
Mfn1
Mfn2
GAPDH
p-Drp1/Drp1
Relative protein level (fold of Control)

Figure S4

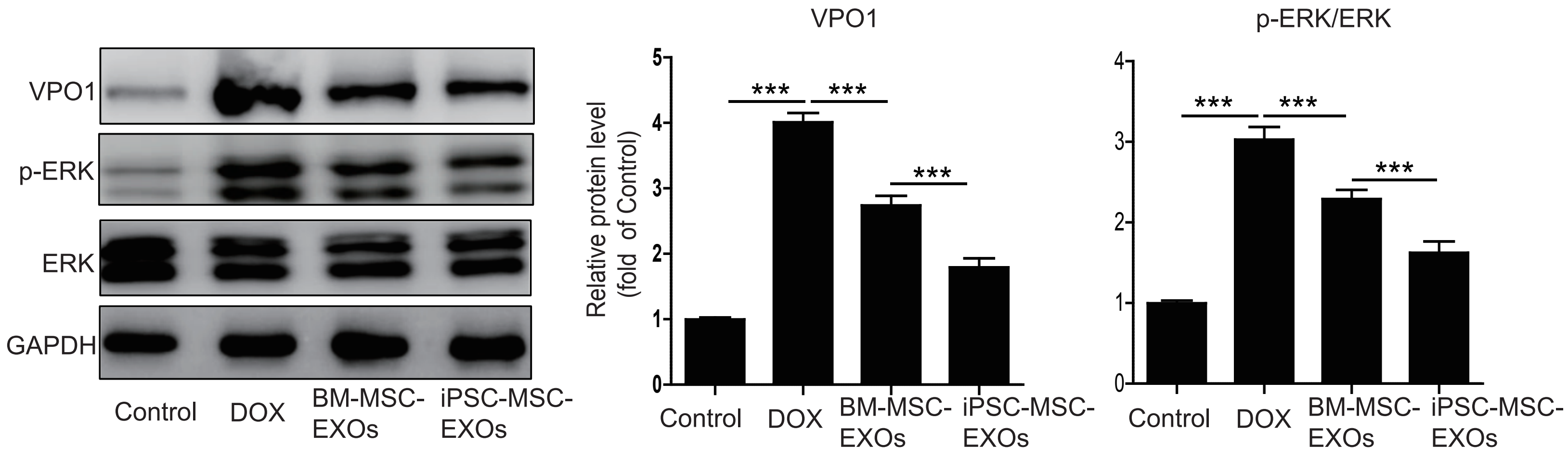
VPO1
p-ERK
ERK
GAPDH
Control
DOX
BM-MSC-EXOs
iPSC-MSC-EXOs
VPO1
Relative protein level (fold of Control)
***
***
***
5
4
3
2
1
0
Control
DOX
BM-MSC-EXOs
iPSC-MSC-EXOs
p-ERK/ERK
***
***
***
4
3
2
1
0
Control
DOX
BM-MSC-EXOs
iPSC-MSC-EXOs

Figure S5

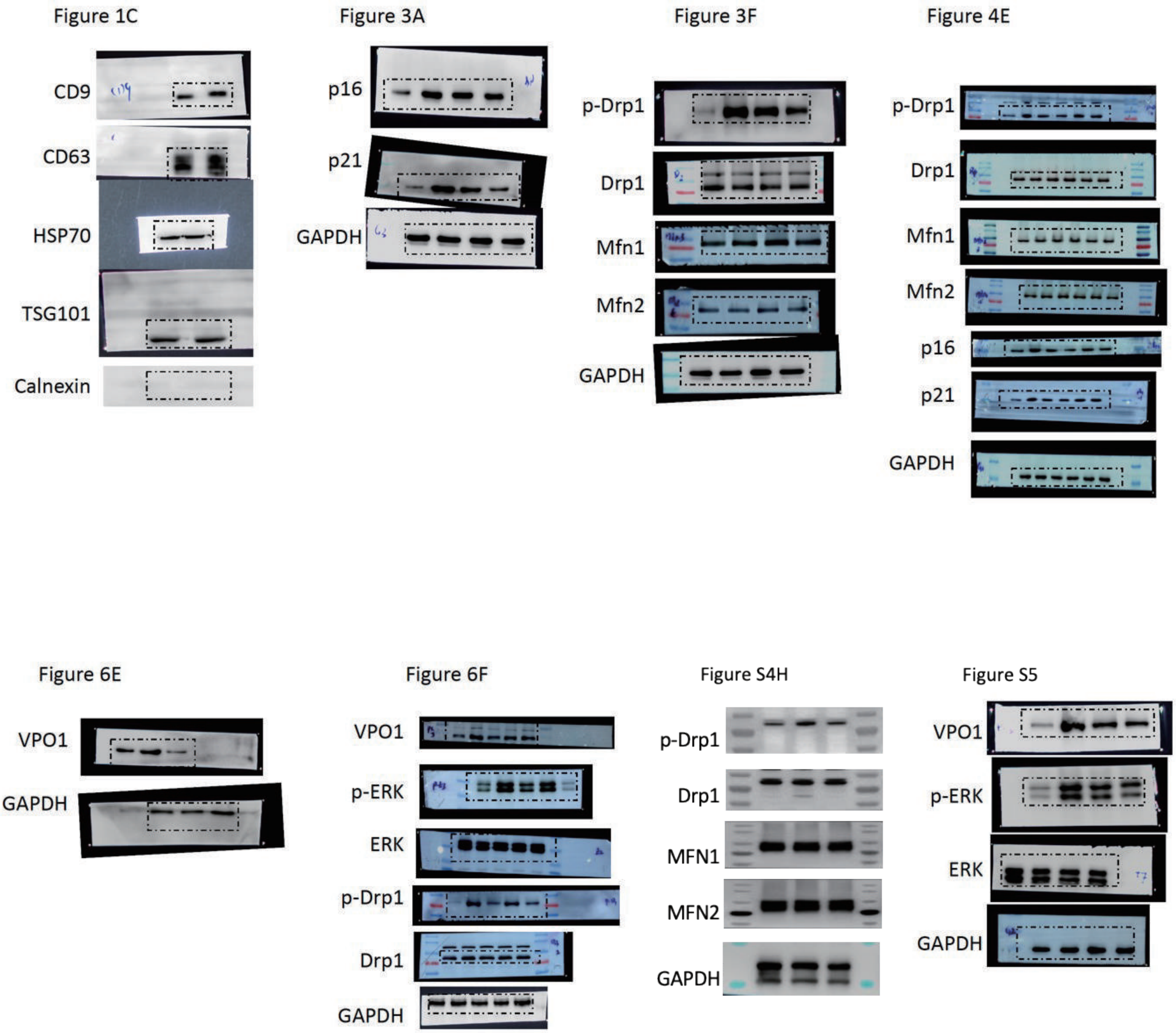
Figure 1C
CD9
CD63
HSP70
TSG101
Calnexin
Figure 3A
p16
p21
GAPDH
Figure 3F
p-Drp1
Drp1
Mfn1
Mfn2
GAPDH
Figure 4E
p-Drp1
Drp1
Mfn1
Mfn2
p16
p21
GAPDH
Figure 6E
VPO1
GAPDH
Figure 6F
VPO1
p-ERK
ERK
p-Drp1
Drp1
GAPDH
Figure S4H
p-Drp1
Drp1
MFN1
MFN2
GAPDH
Figure S5
VPO1
p-ERK
ERK
GAPDH

Figure S6
